# Ovarian Metastasis from Invasive Lobular Carcinoma of the Breast: A 6-Case Series with Emphasis on Diagnostic Challenges and the Value of Biopsy

**DOI:** 10.3390/diagnostics16132019

**Published:** 2026-06-28

**Authors:** Anqi Li, Lei Liu, Dingbao Chen, Yuan Peng, Feng Pan

**Affiliations:** 1 Department of Radiology, Peking University People’s Hospital, Beijing 100044, China (A.L.); (L.L.); (D.C.); 2 Breast Center, Peking University People’s Hospital, Beijing 100044, China

**Keywords:** breast cancer, invasive lobular carcinoma, ovarian metastasis, computed tomography, biopsy, diagnostic errors

## Abstract

**Background**: Invasive lobular carcinoma (ILC) of the breast has a unique metastatic pattern due to E-cadherin deficiency, with a predilection for peritoneal, gastrointestinal, and pelvic organ involvement. Ovarian metastasis from ILC is rare but can mimic primary ovarian cancer clinically and radiologically, leading to misdiagnosis and unnecessary radical surgery. This study aimed to summarize the imaging features of ovarian metastasis from ILC and analyze the causes of misdiagnosis, while highlighting the value of preoperative biopsy. **Methods**: Clinical and imaging data of six patients with pathologically confirmed ovarian metastasis from ILC were retrospectively analyzed. All six patients were female (age range 33–65 years), had a history of breast cancer (ILC subtype), and were found to have ovarian masses either during follow-up or at initial diagnosis. Imaging findings of the ovaries, peritoneum, and ascites were analyzed and compared with initial clinical diagnoses and pathological results. **Results**: Among the six patients, five were initially clinically misdiagnosed as having primary ovarian cancer and underwent unnecessary total hysterectomy with bilateral salpingo-oophorectomy. One patient (case 6) was correctly diagnosed via percutaneous biopsy of the omentum and skin nodules, which confirmed metastatic ILC, thereby avoiding unnecessary gynecological surgery. Imaging findings: All six patients had bilateral ovarian masses, appearing as solid or cystic-solid lesions. Peritoneal changes were observed in four cases. Ascites was present in five cases. Laboratory findings showed marked variability in CA125 levels (normal in three cases, elevated in three cases). Immunohistochemistry confirmed breast origin in all cases (GATA3+, PAX8−, E-cadherin−). In case 6, after four cycles of chemotherapy (albumin-bound paclitaxel + carboplatin + bevacizumab), follow-up CT demonstrated significant reduction in ovarian masses and regression of peritoneal and omental lesions. **Conclusions**: Ovarian metastasis from ILC is easily misdiagnosed as primary ovarian cancer without histologic confirmation, leading to unnecessary radical surgery. However, as demonstrated in case 6, percutaneous biopsy of accessible metastatic sites (omentum, peritoneum, or skin nodules) can establish the correct diagnosis and guide systemic therapy, thereby avoiding unnecessary surgery. In case 6, the ILC ovarian metastasis showed a favorable response to chemotherapy, but this single-case finding requires cautious interpretation as ILC is generally considered less chemosensitive than invasive ductal carcinoma. In patients with a history of breast cancer or with suspected metastatic disease, ILC metastasis should be included in the differential diagnosis of ovarian masses.

## 1. Introduction

Invasive lobular carcinoma (ILC) is the second most common histologic subtype of breast cancer, accounting for approximately 10–15% of all breast cancers [1]. Unlike invasive ductal carcinoma (IDC), ILC exhibits distinct biological behavior characterized by E-cadherin deficiency, which leads to decreased cell adhesion and a single-file infiltrative growth pattern [2]. This unique biology predisposes ILC to metastasize to “atypical” sites, including the peritoneum, gastrointestinal tract, and pelvic organs [3,4,5].

Ovarian metastasis from ILC is relatively rare but clinically significant [6]. Due to the overlapping clinical manifestations, imaging features, and tumor marker profiles, ILC ovarian metastasis is frequently clinically misdiagnosed as primary ovarian cancer, leading to unnecessary radical surgery [7]. The literature on this entity is primarily composed of single case reports, with few case series providing systematic analysis of imaging features from a radiologist’s perspective [8,9].

Here, we report a case series of six patients with pathologically confirmed ovarian metastasis from ILC. We summarize the clinical and imaging characteristics, analyze the causes of misdiagnosis, and highlight the value of percutaneous biopsy in avoiding unnecessary surgery, with one case demonstrating favorable response to systemic chemotherapy.

## 2. Materials and Methods

### 2.1. Study Design and Patient Selection

This retrospective case series was conducted at Peking University People’s Hospital. Six patients with pathologically confirmed ovarian metastasis from ILC were included. Inclusion criteria were (1) pathologically confirmed ILC of the breast; (2) pathological confirmation of ovarian/peritoneal metastasis as breast cancer origin; (3) complete imaging data. Exclusion criteria were primary ovarian cancer or ovarian metastasis from other primary tumors.

### 2.2. Data Collection

Clinical data including age, breast cancer history, treatment history, time interval between breast cancer diagnosis and ovarian metastasis detection, presenting symptoms, laboratory findings (CA125, CA153, CEA), initial clinical diagnosis, and treatment records were collected.

### 2.3. Imaging Protocols

#### 2.3.1. Pelvic Contrast-Enhanced CT

Contrast-enhanced CT examinations were performed on a 256-slice multidetector CT scanner (Revolution CT, GE Healthcare, Milwaukee, WI, USA). Scanning parameters were as follows: tube voltage 120 kVp, automatic tube current modulation, slice thickness 1.25 mm, slice interval 1.25 mm, pitch 0.992, and rotation time 0.5 s. Non-ionic iodinated contrast material (320 mg I/mL;Ultravist, Bayer Schering Pharma, Berlin, Germany) was administered intravenously at a dose of 1.5–2.0 mL/kg body weight with an injection rate of 2.5–3.0 mL/s. Portal venous phase images were acquired at a delay of approximately 60–70 s after contrast injection. All images were reconstructed using a standard soft-tissue kernel.

#### 2.3.2. Pelvic MRI

MRI examinations were performed using 1.5 T (Sigma HDxt) or 3.0 T (Discovery MR750) scanners (GE Healthcare, Milwaukee, WI, USA) with a dedicated pelvic phased-array coil. The protocol included axial T1-weighted imaging (T1WI), axial and sagittal fat-suppressed T2-weighted imaging (T2WI), axial diffusion-weighted imaging (DWI) with b values of 0 and 800 s/mm^2^ (with automatic ADC map generation), and axial and sagittal contrast-enhanced T1-weighted imaging (CE-T1WI). Imaging parameters were as follows: slice thickness 4 mm, slice gap 1 mm, and field of view 24–28 cm. Contrast-enhanced images were acquired after intravenous injection of 0.1 mmol/kg body weight of gadopentetate dimeglumine (Magnevist, Bayer Schering Pharma, Berlin, Germany) at a rate of 2 mL/s, followed by a 20 mL saline flush.

#### 2.3.3. PET-CT

PET-CT examinations were performed on a Discovery 710 scanner (GE Healthcare, Milwaukee, WI, USA). Patients fasted for at least 6 h, and blood glucose levels were confirmed to be <11.1 mmol/L before intravenous injection of 18F-FDG at a dose of 3.7–5.5 MBq/kg body weight. Images were acquired from the skull base to the mid-thigh after an uptake period of approximately 60 min. CT parameters were as follows: tube voltage 120 kVp, slice thickness 3.75 mm. PET emission scans were performed at 2 min per bed position, and images were reconstructed using an iterative algorithm with CT-based attenuation correction. Maximum standardized uptake values (SUVmax) of lesions were calculated on a dedicated workstation.

### 2.4. Image Analysis

Two radiologists with more than 10 years of experience independently reviewed all images. Imaging features assessed included (1) ovarian masses: location (unilateral/bilateral), size, morphology, margin, signal/density characteristics, and enhancement pattern or FDG uptake; (2) peritoneal changes: peritoneal thickening, omental changes, peritoneal nodules; (3) ascites: presence and extent; (4) other findings: lymphadenopathy and metastases to other organs. Disagreements were resolved by consensus.

### 2.5. Pathological Correlation

All patients underwent surgery (*n* = 5) or biopsy (*n* = 1). Immunohistochemistry (IHC) was performed for GATA3, PAX8, ER, PR, E-cadherin, CK7, and CK20.

### 2.6. Ethical Considerations

This retrospective study was approved by the Institutional Review Board of Peking University People’s Hospital (Approval No. 2026PHB157-001 and date of approval 4 February 2026). Informed consent was waived due to the retrospective nature of the study.

## 3. Results

### 3.1. Clinical Characteristics

The clinical characteristics of the six patients are summarized in Table 1. All six patients were female with a median age of 44 years (range 33–65 years). All patients had pathologically confirmed ILC. The time interval from breast cancer diagnosis to ovarian metastasis detection ranged from 0 (synchronous) to 13 years.

### 3.2. Imaging Findings

The imaging findings of the six patients are summarized in Table 2. Ovarian masses were present in all six cases (100%), and all were bilateral. Peritoneal changes were observed in four cases (67%). Ascites was present in five cases (83%). FDG uptake on PET-CT was documented in two patients (cases 2 and 5), with a maximum standardized uptake value (SUVmax) ranging from 3.2 to 4.9. Pelvic ultrasound in all six cases demonstrated findings consistent with CT and MRI: predominantly solid hypoechoic adnexal masses with irregular margins and internal vascularity in cases 2, 3, 4, and 6; and complex cystic-solid lesions with internal echogenic debris and vascularized solid components in cases 1 and 5.

**Figure 1:**
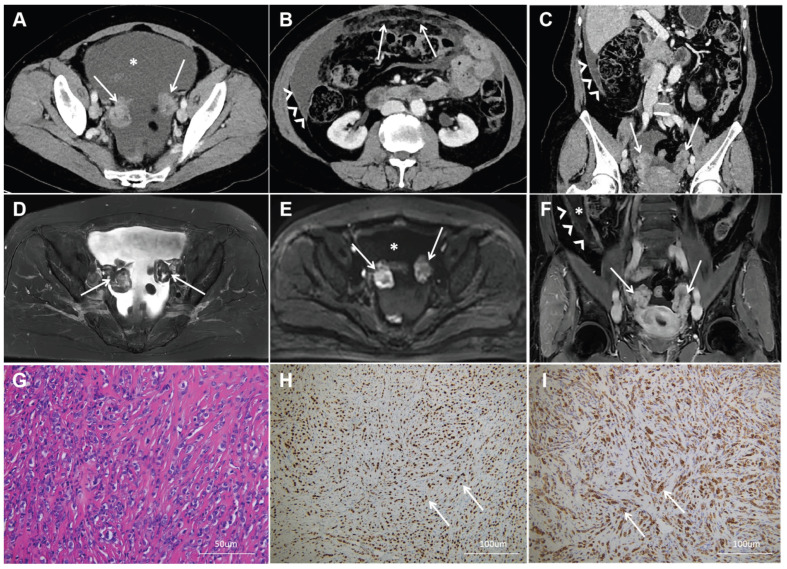
Case 4, a woman with right ILC 13 years after breast cancer surgery, presenting with abdominal discomfort. (**A**) Contrast-enhanced CT shows bilateral solid adnexal nodules (arrows); (**B**) axial contrast-enhanced CT shows omental cake (arrows) and miliary peritoneal nodules (arrowheads), along with massive ascites; (**C**) coronal contrast-enhanced CT shows miliary peritoneal nodules (arrowheads), and bilateral solid adnexal nodules (arrows); (**D**) axial T2WI shows bilateral solid adnexal nodules (arrows); (**E**) axial DWI (b = 800 s/mm^2^) shows hyperintensity of the bilateral adnexal nodules; axial contrast-enhanced T1WI shows marked heterogeneous enhancement of the bilateral adnexal nodules (arrows); (**F**) coronal contrast-enhanced T1WI shows miliary peritoneal nodules (arrowheads), and bilateral solid adnexal nodules (arrows); (**G**) hematoxylin and eosin (HE) staining of the ovarian metastasis shows diffuse infiltration of tumor cells with a characteristic single-file (Indian file) pattern (original magnification ×200); (**H**) immunohistochemistry for ER shows strong nuclear positivity in tumor cells (arrows) (original magnification ×100); (**I**) immunohistochemistry for CK7 shows cytoplasmic positivity in tumor cells (arrows) (original magnification ×100). * in (**A**,**E**,**F**) indicate ascites.

**Figure 2:**
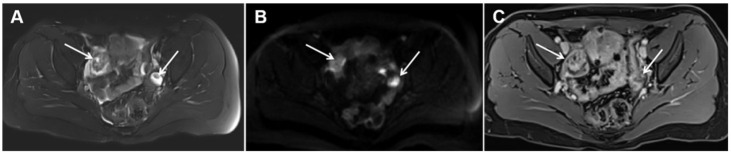
Case 1, a woman with right ILC 2 years after breast cancer surgery, asymptomatic on routine screening. (**A**) Axial T2WI shows bilateral ovarian cystic-solid masses (arrows); (**B**) axial DWI (b = 800 s/mm^2^) shows hyperintensity of ovarian masses (arrows); (**C**) axial contrast-enhanced T1WI shows enhancement of the solid components of the ovarian masses (arrows).

**Figure 3:**
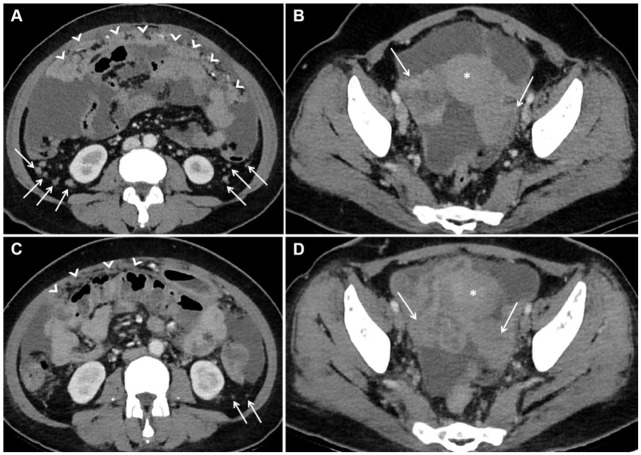
Case 6: Axial contrast-enhanced CT images before and after chemotherapy. (**A**) Before treatment, omental cake (arrowheads) and extensive peritoneal and retroperitoneal nodules (arrows) are visible. (**B**) Before treatment, a right cystic-solid ovarian mass and a left solid ovarian mass (arrows) are present; the uterus is marked by an asterisk. (**C**) After 4 cycles of chemotherapy, the omental cake has markedly regressed (arrowheads) and the retroperitoneal nodules have nearly disappeared (arrows). (**D**) After 4 cycles of chemotherapy, the right cystic-solid ovarian mass has significantly reduced in size, the left solid ovarian mass has mildly reduced (arrows), and the uterus remains visible (asterisk).

Case 4 demonstrated the most extensive peritoneal involvement. CT and MRI showed bilateral solid ovarian masses with contrast enhancement, accompanied by prominent omental cake, miliary peritoneal nodules, and massive ascites (Figure 1A–F). Case 1 presented with bilateral ovarian cystic-solid masses that appeared hyperintense on DWI and showed marked enhancement of the solid components on contrast-enhanced T1WI (Figure 2A–C). Case 6 had bilateral solid ovarian masses with diffuse peritoneal and omental nodules at baseline (Figure 3A,B). After four cycles of chemotherapy, follow-up CT demonstrated reduction in the ovarian masses and regression of the peritoneal and omental nodules (Figure 3C,D).

### 3.3. Misdiagnosis

Among the six patients, five were initially misdiagnosed as having primary ovarian cancer and underwent total hysterectomy with bilateral salpingo-oophorectomy (cases 1–5). In contrast, case 6 was correctly diagnosed via percutaneous biopsy of the omentum and skin nodules, which confirmed metastatic ILC. This patient did not undergo gynecological surgery and received systemic chemotherapy instead.

### 3.4. Pathology and Immunohistochemistry

All six patients had pathologically confirmed ovarian/peritoneal metastasis from ILC. Immunohistochemistry showed GATA3 positivity, PAX8 negativity, and E-cadherin negativity in all cases, confirming breast origin. Representative histopathological images from case 4 (HE staining, ER, and CK7) are shown in Figure 1G–I. ER/PR expression varied across cases, with case 3 showing loss of ER/PR expression compared to the primary tumor. To provide a comprehensive overview of receptor status heterogeneity between primary and metastatic sites, we summarized the ER, PR, and HER2 expression of both the primary breast tumor and the corresponding ovarian metastasis for all six cases in Table 3.

### 3.5. Treatment and Follow-Up

Treatment and follow-up data for all six cases are summarized in Table 4. Cases 1–5 underwent total hysterectomy with bilateral salpingo-oophorectomy, followed by endocrine therapy or chemotherapy. Case 6 received chemotherapy with albumin-bound paclitaxel + carboplatin + bevacizumab. After four cycles of chemotherapy, follow-up CT demonstrated reduction in ovarian masses (left: 4.5 cm → 3.8 cm; right: 3.9 cm → 2.7 cm) and regression of peritoneal and omental nodules. The patient continued chemotherapy and had no evidence of progression at 5 months of follow-up. Follow-up outcomes varied among the surgically treated patients: Cases 2, 4 and 6 were alive with disease, Case 3 was lost to follow-up after brain metastasis, and Cases 1 and 5 remained recurrence-free (Table 4).

## 4. Discussion

### 4.1. Clinical Characteristics of ILC Ovarian Metastasis

ILC is characterized by E-cadherin deficiency, which results in reduced cell adhesion and facilitates single-file infiltration and distant metastasis [1]. Unlike IDC, ILC preferentially metastasizes to serosal surfaces including the peritoneum, gastrointestinal tract, and ovaries [10,11].

Our case series revealed several representative clinical features—relatively young age (33–65 years), which is noteworthy as ILC typically affects older women. This may be explained by tertiary referral center bias, earlier detection through active surveillance, or potential genetic predisposition—although BRCA testing was not performed in this retrospective cohort and is acknowledged as a limitation—consistent with the wide age range reported in the literature. A prolonged and highly variable interval from primary diagnosis to ovarian metastasis (0–13 years) indicates that ILC can recur at an exceptionally late stage, far beyond the typical 5-year surveillance window, which strongly supports the need for extended long-term surveillance, particularly in hormone receptor-positive patients [4] and diverse modes of presentation, including asymptomatic detection (cases 1 and 5), abdominal symptoms (case 4), elevated tumor markers (cases 2 and 3), and synchronous presentation with primary breast cancer (case 6). Notably, case 6 presented with bilateral ovarian and peritoneal metastases as the initial manifestation of previously undiagnosed breast cancer, a scenario that has been reported as “occult breast cancer presenting as ovarian metastases”.

### 4.2. Imaging Features of ILC Ovarian Metastasis

Our study identified the following imaging features of ILC ovarian metastasis. Regarding ovarian masses, all were bilateral (6/6, 100%). The masses appeared solid or cystic-solid, with contrast enhancement or FDG uptake. Cases 2 and 5 demonstrated FDG uptake on PET-CT. These features overlap with those of primary ovarian cancer, making radiological differentiation challenging [12].

Regarding peritoneal changes, four cases (67%) showed peritoneal involvement, including omental cake, peritoneal thickening, and miliary nodules. Case 4 and 6 exhibited the most typical findings, with extensive peritoneal thickening and miliary nodules, indistinguishable from peritoneal dissemination of primary ovarian cancer. This pattern of peritoneal carcinomatosis is a well-recognized manifestation of metastatic ILC [5,13]. Despite this recognition, the imaging findings in our patients were initially interpreted as primary ovarian cancer rather than metastatic ILC. Several factors contributed to this misdiagnosis: the imaging features overlap significantly with those of primary ovarian cancer; the long interval between primary breast cancer diagnosis and ovarian metastasis detection (up to 13 years in Case 4) led clinicians to consider a second primary rather than a late recurrence; markedly elevated CA125 in some cases (e.g., 425 U/mL in Case 4) strongly suggested primary ovarian cancer; and clinicians may have limited familiarity with the distinctive metastatic behavior of ILC, which preferentially involves serosal surfaces including the peritoneum and ovaries.

Regarding ascites, ascites was present in five cases (83%), ranging from mild to massive. In case 4, massive ascites accompanied by significantly elevated CA125 and human epididymis protein 4 (HE4) strongly suggested primary ovarian cancer.

The imaging features of ILC ovarian metastasis are notoriously non-specific. A study on MRI diagnosis of ovarian malignancies noted that metastatic tumors often present with solid components and bilateral involvement, features that overlap with primary epithelial ovarian cancer [14]. This diagnostic ambiguity underscores the need for a high index of suspicion in patients with a history of breast cancer, particularly the lobular subtype.

### 4.3. Association Between Ovarian and Gastrointestinal Metastases

Notably, two of our six patients (cases 2 and 3, 33%) developed gastric metastases either simultaneously with or subsequent to ovarian metastasis. This phenomenon can be explained by the “intraperitoneal seeding” hypothesis: E-cadherin-deficient ILC cells readily detach from the primary tumor, enter the peritoneal cavity, and implant on peritoneal surfaces, including the omentum, ovaries, and gastrointestinal serosa [15,16].

The predilection of ILC for gastrointestinal metastases has been well documented. A recent comprehensive review demonstrated that ILC has a distinct metastatic pattern with notable predilection for gastrointestinal involvement, including gastric and colorectal metastases [16]. The small, discohesive ILC cells infiltrate the gastric wall in a diffuse, linitis plastica-like pattern, often without forming a discrete mass, making endoscopic detection challenging. In case 3, gastric wall thickening was detected on CT and subsequently confirmed by biopsy, highlighting the importance of radiographic surveillance of the gastrointestinal tract in patients with known ILC metastases.

For patients with ILC ovarian metastasis, enhanced surveillance of the digestive tract is recommended. Initial imaging should carefully evaluate gastric and intestinal wall thickening. Upper endoscopy should be considered when gastrointestinal symptoms develop, and serial monitoring of CA153 and CA199 levels may provide early clues for gastrointestinal involvement.

### 4.4. The Critical Role of Immunohistochemistry in Differential Diagnosis

One of the most important lessons from our series is that histopathological examination is the gold standard for distinguishing ILC ovarian metastasis from primary ovarian cancer. ILC is characterized by single-file (Indian file) infiltration of small, discohesive cells with minimal desmoplastic reaction. However, when the characteristic growth pattern is not well represented—particularly in small biopsy specimens—morphology alone may be equivocal. In such challenging cases, immunohistochemistry plays a critical supplementary role. Key markers include GATA3, GCDFP-15, ER/PR, PAX8, WT1, and E-cadherin. In ILC metastasis, GATA3 is positive, PAX8 is negative, WT1 is negative, and E-cadherin expression is lost; in contrast, primary ovarian cancer typically shows PAX8 positivity, WT1 positivity (in serous types), and retained E-cadherin expression. The combination of GATA3 positivity, PAX8 negativity, and loss of E-cadherin expression definitively confirms breast origin.

A case report of occult lobular breast carcinoma presenting as bilateral ovarian masses emphasized that immunohistochemical profiling is “essential in identifying breast origin in metastatic ovarian tumors” [17]. The authors noted that GATA3 and GCDFP-15 positivity, combined with PAX8 negativity, is highly specific for breast origin. In all our cases, the ovarian metastases demonstrated GATA3 positivity, PAX8 negativity, and loss of E-cadherin expression, definitively confirming their breast origin.

A notable finding in our series is case 3, where the primary breast tumor was ER/PR strongly positive, but the ovarian metastasis showed complete loss of ER/PR expression. This phenomenon of receptor status conversion in metastatic sites is well recognized and has important therapeutic implications. Patients with receptor-negative metastases may not benefit from endocrine therapy and may require alternative treatment strategies such as chemotherapy or targeted therapy [18]. This finding underscores the importance of biopsying metastatic lesions to guide treatment decisions, rather than relying solely on the primary tumor’s receptor profile.

### 4.5. Misdiagnosis and the Value of Percutaneous Biopsy

Several factors contribute to the high misdiagnosis rate. The first is overlapping clinical manifestations: both ILC ovarian metastasis and primary ovarian cancer can present with pelvic masses, abdominal distension, and ascites. The second is misleading tumor markers: CA125 levels showed marked variability in our series—normal in cases 1, 3, and 5; transiently mild elevation in case 2; and marked elevation in cases 4 (425 U/mL) and 6 (598 U/mL). Therefore, normal CA125 does not exclude ILC ovarian metastasis, and significantly elevated CA125 alone does not confirm primary ovarian cancer [19,20]. The third is similar imaging features: as discussed, CT, MRI, and PET-CT features overlap significantly. The fourth is lack of awareness of ILC biology: clinicians and radiologists may be unfamiliar with the unique metastatic pattern of ILC, failing to associate a history of breast cancer with an ovarian mass, especially when the time interval is long (up to 13 years in case 4).

Case 6 provides a compelling argument for obtaining tissue diagnosis before proceeding with surgery. The patient presented with synchronous breast cancer and widely metastatic disease involving the ovaries, peritoneum, omentum, and skin. Rather than proceeding with diagnostic laparotomy, a percutaneous biopsy of an easily accessible metastatic site (omentum and skin nodules) was performed. This minimally invasive procedure established the correct diagnosis of metastatic ILC, sparing the patient an unnecessary total hysterectomy with bilateral salpingo-oophorectomy, and allowed for immediate initiation of systemic chemotherapy. The literature supports this approach. A case report of a 38-year-old woman with invasive lobular carcinoma diagnosed by inguinal lymph node biopsy demonstrated that core needle biopsy of an accessible metastatic site can establish the diagnosis and guide therapy, avoiding more invasive procedures [21]. Similarly, a study on management of ovarian metastasis from lobular breast carcinoma emphasized that surgical evaluation should be performed only when imaging is suspicious and CA125 is elevated, suggesting that a selective approach can reduce unnecessary surgeries [22].

### 4.6. Response to Systemic Therapy and Prognosis

Case 6 also demonstrated a favorable response to chemotherapy (albumin-bound paclitaxel plus carboplatin plus bevacizumab), with imaging-confirmed reduction in ovarian masses (left from 4.5 cm to 3.8 cm; right from 3.9 cm to 2.7 cm) and regression of peritoneal and omental nodules after only four cycles. We acknowledge that this single-case observation has a short follow-up period (5 months) and should not be generalized, as ILC is generally considered less chemosensitive than invasive ductal carcinoma. Nevertheless, this case highlights that accurate diagnosis via biopsy can guide systemic therapy and potentially avoid unnecessary surgery in selected patients, while therapeutic decisions should continue to be guided by established guidelines and higher-quality evidence.

The literature provides additional evidence for chemosensitivity in metastatic ILC [23]. A comprehensive review on invasive lobular breast cancer highlighted that, despite the distinct biological behavior of ILC characterized by E-cadherin deficiency and single-file infiltration, metastatic ILC shows responsiveness to systemic therapies, including chemotherapy and endocrine therapy, with outcomes comparable to those of invasive ductal carcinoma in certain settings [23]. These findings, together with the favorable treatment response observed in our case 6, suggest that systemic therapy can be highly effective in ILC ovarian metastases, potentially obviating the need for surgery.

Regarding long-term prognosis in our series, the outcomes demonstrated significant heterogeneity. Case 1 had no recurrence at 21 months of follow-up after endocrine therapy. Case 2 developed lymph node, bone, and gastric metastases nine years after ovarian surgery. Case 3 developed brain metastasis three months after ovarian surgery and was lost to follow-up. Case 5 had no recurrence at 10 years of follow-up on letrozole. Case 6 achieved partial response at five months of ongoing chemotherapy. This variability likely reflects differences in tumor biology, including proliferation index and hormone receptor status. Notably, case 3, which had the highest Ki67 (67%) and loss of ER/PR expression, had the poorest outcome, developing brain metastases within three months. This observation aligns with the literature suggesting that loss of hormone receptor expression in metastatic sites is associated with more aggressive behavior and worse prognosis [24,25].

### 4.7. Clinical Implications and Recommendations

Our findings have several practical implications for the management of patients with suspected ILC ovarian metastasis.

For radiologists, we recommend maintaining a high index of suspicion for ILC metastasis when interpreting pelvic imaging in patients with a history of breast cancer, particularly lobular subtype. Radiologists should carefully evaluate for associated findings: bilateral ovarian masses, peritoneal thickening or nodules, omental caking, and ascites. When these findings are present, we suggest raising the possibility of metastatic ILC in the radiology report and recommending biopsy for confirmation. If the patient has no prior history of breast cancer but imaging suggests metastatic disease to the ovaries (e.g., bilateral solid masses with peritoneal involvement), radiologists should consider the breast as a possible primary site and recommend breast imaging. We acknowledge that O-RADS risk stratification was not applicable to our retrospective cohort, as this system had not been implemented in our institutional practice at the time of image interpretation. Future prospective studies incorporating standardized O-RADS reporting would facilitate more consistent assessment and comparison of ovarian masses in patients with a history of ILC [26].

For oncologists and surgeons, it is important to recognize that ILC has a unique metastatic pattern with predilection for the peritoneum and ovaries. In patients with a breast cancer history presenting with adnexal masses, clinicians should not automatically assume a new primary ovarian cancer. A study found that the risk of developing an ovarian neoplasm is higher in women with a history of breast cancer [27,28]. However, when malignant, metastatic breast cancer should be strongly considered. Before proceeding with radical gynecologic surgery, we recommend obtaining tissue diagnosis via percutaneous biopsy of an accessible site (omentum, lymph node, or ascites) whenever possible. If biopsy confirms metastatic ILC, systemic therapy (endocrine therapy, chemotherapy, or targeted therapy) should be initiated based on the receptor profile of the metastatic lesion, which may differ from the primary tumor.

For pathologists, when evaluating ovarian masses in patients with a breast cancer history, or even without a known primary, we recommend performing a panel of immunohistochemical stains including GATA3, PAX8, ER, PR, and E-cadherin. The combination of GATA3 positivity, PAX8 negativity, and loss of E-cadherin expression is diagnostic of metastatic ILC [29]. Additionally, we recommend reporting the receptor status (ER, PR, HER2) of the metastatic lesion, as this may guide systemic therapy.

The complex diagnostic challenges posed by ILC ovarian metastases underscore the value of multidisciplinary tumor boards. Radiologists, oncologists, surgeons, and pathologists must collaborate to review imaging, discuss differential diagnoses, and plan the most appropriate diagnostic and therapeutic approach. Case 6 exemplifies the benefits of this approach: the diagnosis was made by biopsy, avoiding surgery, and systemic therapy was initiated promptly under the guidance of a multidisciplinary team.

### 4.8. Study Limitations

Several limitations of this study should be acknowledged. First, the sample size is small (six cases), which limits the generalizability of our findings. Second, this is a retrospective case series, which may introduce selection bias. Third, the imaging modalities were not uniform across all cases. Fourth, original images were not available for all cases. Fifth, follow-up durations varied among cases. Sixth, as a single-center study, our findings may not be representative of broader populations. Future multicenter prospective studies with larger sample sizes and standardized imaging protocols are needed to validate our findings and establish more definitive imaging criteria for differentiating ILC ovarian metastasis from primary ovarian cancer.

## 5. Conclusions

Ovarian metastasis from invasive lobular carcinoma of the breast is easily misdiagnosed as primary ovarian cancer due to overlapping imaging features and tumor marker profiles, leading to unnecessary radical gynecological surgery, as occurred in five of our six patients. Imaging findings—bilateral solid ovarian masses with peritoneal changes and ascites—are non-specific, and CA125 levels are unreliable. However, as demonstrated by case 6, percutaneous biopsy of accessible metastatic sites (omentum, peritoneum, or skin nodules) can establish the correct diagnosis, prevent unnecessary surgery, and guide effective systemic therapy, with imaging-confirmed treatment response. We recommend that tissue biopsy be pursued before radical surgery in patients with a history of breast cancer, particularly the lobular subtype, presenting with ovarian masses. However, this recommendation must be interpreted with caution: image-guided biopsy is generally reserved for safely accessible lesions, particularly extra-ovarian sites such as peritoneal implants or nodal metastases. Biopsy of isolated ovarian masses may be technically challenging and carries potential risks, including tumor rupture, disease dissemination, and procedural complications. Therefore, the decision to biopsy should be made on a case-by-case basis, weighing the diagnostic benefits against the procedural risks.

## Figures and Tables

**Table 1:** Clinical characteristics of six patients with ovarian metastasis from ILC.

Characteristic	Case 1	Case 2	Case 3	Case 4	Case 5	Case 6
Age (years)	44	42	33	65	44	44
Breast cancer history	Right ILC	Bilateral ILC	Left ILC	Right ILC	Left ILC	Bilateral ILC
Time interval (years)	2	4	8	13	4	0 (synchronous)
CA125 (U/mL)	Normal	55.4	Normal	425	Normal	598
CA153 (U/mL)	Normal	36.96	40.82	57.7	Normal	300
Initial clinical diagnosis	Primary ovarian cancer	Primary ovarian cancer	Possible ovarian cancer	Ovarian cancer with peritoneal metastasis	Primary ovarian cancer	Metastatic breast cancer
Gynecological surgery performed	Yes	Yes	Yes	Yes	Yes	No
Misdiagnosed	Yes	Yes	Yes	Yes	Yes	No

**Table 2:** Imaging findings of 6 patients with ovarian metastasis from ILC.

Finding	Case 1	Case 2	Case 3	Case 4	Case 5	Case 6
Imaging modality	Pelvic MRI	PET-CT	Pelvic CT	Pelvic CT + MRI	PET-CT	Pelvic CT
Ovarian mass (R/L, cm)	Bilateral (3.1/1.5)	Bilateral (4.2/9.1)	Bilateral (9.0/3.2)	Bilateral (3.6/3.1)	Bilateral (4.0/5.2)	Bilateral (4.5/3.9)
Mass characteristics	Solid-cystic	Solid	Solid	Solid	Solid-cystic	Solid
Peritoneal changes	None	miliary nodules	miliary nodules	Omental cake, miliary nodules	None	Diffuse peritoneal nodules and omental cake
Ascites	Mild	None	Mild	Massive	Mild	Moderate
Lymph nodes	None	Retroperitoneal	Retroperitoneal	Axillary	None	Axillary
Other metastases	None	Gastric, bone	Gastric, brain	None	None	Skin, breast, brain, liver

**Table 3:** Estrogen receptor (ER), progesterone receptor (PR), and HER2 status in primary breast tumors and corresponding ovarian metastases.

Case	Primary Tumor (ER/PR/HER2)	Ovarian Metastasis (ER/PR/HER2)
1	+/+/−	+/+/−
2	+/+/−	+/+/−
3	+/+/−	−/−/−
4	+/+/−	+/+/−
5	+/+/−	+/+/−
6	+/+/−	+/+/−

**Table 4:** Treatment and follow-up data of 6 patients with ovarian metastasis from ILC.

Case	Surgery (TH + BSO)	Chemotherapy	Endocrine Therapy	Targeted Therapy	Follow-Up Duration	Recurrence/Progression	Outcome
1	Yes	No	Yes	No	21 months	No	No recurrence
2	Yes	Yes	Yes	No	9 years	Yes (lymph node, bone, gastric)	Alive with disease
3	Yes	Yes	No	No	3 months	Yes (brain)	Lost to follow-up
4	Yes	Yes	Yes	No	3 months	Yes (lymph node)	Alive with disease
5	Yes	No	Yes	No	10 years	No	No recurrence
6	No	Yes	No	Yes	5 months	No (partial response)	Alive with disease

TH + BSO, total hysterectomy with bilateral salpingo-oophorectomy.

## Data Availability

The original contributions presented in this study are included in the article. Further inquiries can be directed to the corresponding author.
